# Quantitative Analysis of Volatile Impurities in Diallyldimethylammonium Chloride Monomer Solution by Gas Chromatography Coupled with Liquid-Liquid Extraction

**DOI:** 10.1155/2017/6041459

**Published:** 2017-01-23

**Authors:** Cheng Liu, Yuejun Zhang, Haiying Wang, Weixin Wang

**Affiliations:** School of Chemical Engineering, Nanjing University of Science & Technology, Nanjing 210094, China

## Abstract

The quantitative analysis method for volatile impurities in diallyldimethylammonium chloride (DADMAC) monomer solution was established in this paper. The volatile impurities were quantitatively analyzed with trichloromethane as extraction solvent and n-hexane as internal standard by using gas chromatography (GC) coupled with solvent extraction, and the chromatographic conditions, quantitative methods, and extraction conditions were systematically investigated in detail. The results showed that excellent linear relationships of 5 volatile impurities (dimethylamine, allyldimethylamine, allyl chloride, allyl alcohol, and allyl aldehyde) were obtained in the range of 1–100 mg·L^−1^. The method also showed good specificity, recovery (95.0%–107.5%), and relative standard deviation (RSD, 1.40%–7.67%). This method could accurately detect the whole volatile impurities in DADMAC monomer solution quantitatively in one time with a low detection limit. Furthermore, this method is conducive to the preparation of highly pure DADMAC monomer and the development of national and international standards of the DADMAC monomer product quality, and the results could provide a strong foundation for the regulation and mechanism research of impurities on monomer reactivity in polymerization.

## 1. Introduction

Diallyldimethylammonium chloride (DADMAC) is a kind of quaternary ammonium monomer containing two unsaturated double bonds [1, 2] and it usually exists as aqueous solution because of strong polarity and hygroscopic property [3]. DADMAC is a very technologically important monomer and linear polymers of DADMAC can be obtained according to Butler's cyclopolymerization protocol [4] which have been an attractive method for the polymerization of diallylammonium monomers or their copolymerizations [5]. Homo- and copolymers of DADMAC are most widely used cationic water-soluble polymers in the fields of petroleum [6], dyeing [7], paper making [8], daily chemicals [9], and water treatment [10–12] because of good water solubility, easy-controlled positive charge density and molecular weight, and nontoxicity in wide range of pH and temperature.

As for the DADMAC-based cationic polyelectrolyte with linear structure, the application fields and efficiency are determined by the molecular weight and distribution [13] when the cationic unit structure and its ratio in a polymer chain are fixed. As a result, the research on the products with high and serial molecular weight or the products with controlled molecular weight distribution is always the key point [14]. However, for DADMAC-based polymers formed from radical polymerization, the species, and amounts of impurities in DADMAC monomer solution have been considered to be one of the key influencing factors on the polymerization and product molecular weights, in addition to the polymerization technology [15], the sorts and amounts of initiators [16], and so on. Usually, the impurities existing in DADMAC monomer solution include residual raw materials, intermediate products, and byproducts [17]. The known studies showed that monomers with different purification levels had different polymerization reactivity at the same reaction condition [18]. And in particular the intrinsic viscosity of the homopolymer of DADMAC dropped rapidly after adding impurity components such as intermediate product allyldimethylamine and byproduct allyl alcohol into the monomer solution purified by recrystallization [19]. Furthermore, the impurities that remained in the polymer products might be transformed into toxic and harmful byproducts interfering the application performance of polymers [20, 21].

The qualitative and quantitative analysis of impurities in DADMAC monomer solution is the basic mean for accurately controlling impurities. In the early 1960s, Boothe [22] tested the monomers from different purification processes by gas chromatography and the results showed that the peak numbers and strength of unknown impurity components decreased obviously till disappearance. Recently, Wang et al. [23] transformed the amine salts into volatile organic amines by adjusting pH with alkali and then measured the absorbed amines in water by titration to determine the total amounts of amine-impurities in DADMAC monomer solution. However, it had failed not only to determine the types and contents of amines and amine salts, but also to concern other impurities.

In this study, based on the formation mechanism of impurities in synthesis process of DADMAC monomer, we reported the development and validation of a new method using gas chromatography (GC) coupled with solvent extraction to detect impurities in DADMAC monomer solution. This quantitative method could forcefully support the research on preparation technology for high purity DADMAC monomer, the establishment of DADMAC product quality standard, and the study on the mechanism and regulation concerning effects of impurities on monomer polymerization reactivity.

## 2. Materials and Methods

### 2.1. Chemicals

Dimethylamine (≥99.8%), allyldimethylamine (>99.0%), allyl chloride (>99.8%), allyl alcohol (>99.5%), and allyl aldehyde (>99.0%) were purchased from Sigma-Aldrich (USA). Reagents (methanol, ethanol, 1-propanol, isopropanol, n-hexane, acetone, dichloromethane, trichloromethane, and ethyl acetate) were of analytical grade and purchased from Sinopharm Chemical Reagent Co., Ltd. (China). Double distilled water was used throughout.

According to [24], standard solution of DADMAC monomer (60 wt%) was prepared by dissolving DADMAC crystal into double distilled water.

### 2.2. Instrument and Analytical Conditions

A Bruker GC450 gas chromatograph (USA) equipped with a flame ionization detector (FID) was used for quantification of volatile impurities. Three columns, including SE-54 (5% phenyl-95% dimethyl siloxane), PEG-20M (polyethylene glycol), and alkali-modified PEG-20M capillary columns (30 m × 0.25 mm, 0.25 *μ*m), were used for the analysis processes. The temperatures of the injection port and detector were 150 and 250°C, respectively. The oven temperature began at 40°C, held for 2.0 min, and increased at a rate of 10°C·min^−1^ to 120°C. Nitrogen was used as carrier gas at a rate of 20 mL·min^−1^. A volume of 0.2 *μ*L was injected manually in splitless mode.

### 2.3. Preparation of Standard Solutions

Stock solutions of the analysis standards and internal standard (*n*-hexane) were prepared by dissolving them in trichloromethane for final concentration of 1000 mg·L^−1^. A series of working solutions were prepared by diluting the stock solutions to 1, 5, 10, 20, 50, and 100 mg·L^−1^ with trichloromethane; meanwhile internal standard solution was added into the working solutions with final concentration of 20 mg·L^−1^.

### 2.4. Preparation of Samples

The tested DADMAC monomer solution (10 mL, 60 wt%) with impurities was placed in a 25 mL separatory funnel, and then a volume of 10 mL extraction solvent trichloromethane was added into the solution by a pipette. The mixture was shaken for 2 min, then stood for several minutes and demixed. The extraction phase was isolated out from the funnel, and dried by anhydrous sodium sulfate-silica gel. 0.1 mL internal standard solution was added into 4.0 mL of the extraction phase and then the solution was diluted to 5.0 mL by trichloromethane for GC analysis.

In order to investigate the extraction efficiency, standard solution of DADMAC monomer was prepared by adding appropriate amounts of analysis standards and then processed as aforementioned solvent extraction procedure. Extraction efficiency (*R*) could be calculated as follows:

(1) $$\begin{array}{c} R=\frac{A_{o}/A_{Standard}-A_{i}/A_{Standard}}{A_{o}/A_{Standard}}\times 100\%, \end{array}$$

where *A*_*o*_/*A*_Standard_ and *A*_*i*_/*A*_Standard_ were the peak area ratios of impurities versus internal standard before and after extraction. The arithmetic mean of extraction efficiencies at concentrations of 10, 50, and 90 mg·L^−1^ was defined as the extraction efficiency of the impurity in the solvent extraction procedure.

## 3. Results and Discussion

### 3.1. Formation of Volatile Impurities

DADMAC monomer was prepared by the reaction of dimethylamine with allyl chloride and sodium hydroxide, and the general reaction equation was as follows:

(2) $$\begin{array}{c} 2CH_{2}=CHCH_{2}Cl+{\left(\;CH_{3}\;\right)}_{2}NH+NaOH⟶\\ {\left(\;CH_{2}=CHCH_{2}\;\right)}_{2}N^{+}{\left(\;CH_{3}\;\right)}_{2}{Cl}^{-}+NaCl+H_{2}O \end{array}$$

The whole reaction process could be divided into two steps, including alkylation of dimethylamine and the quaternarization. Alkylation of dimethylamine referred to S_N_2 nucleophilic substitution reaction of allyl chloride with nucleophilic reagent dimethylamine [9]. The Cl atom in allyl chloride was very active to be substituted, and dimethylamine with strong alkality (p*K*_*b*_ = 3.27) was also a strong nucleophilic agent [25], so this reaction could be carried out at low temperature. But it was also an exothermic reaction and cooling was essential; otherwise problems such as more side reactions and loss of volatile raw materials would appear. The general equation of alkylation was as follows:

(3) CH32NH+CH2=CHCH2Cl⟶CH2=CHCH2NCH32+HCl

Quaternarization referred to the formation of quaternary ammonium salt by the reaction of allyldimethylamine with allyl chloride [3]. This reaction was essentially also a S_N_2 nucleophilic substitution reaction. The nucleophilic reagent was allyldimethylamine with weaker alkality and stronger steric effect than dimethylamine, so this reaction needed a higher temperature [17]. The general equation of alkylation was as follows:

(4) $$\begin{array}{c} CH_{2}=CHCH_{2}N{\left(\;CH_{3}\;\right)}_{2}+CH_{2}=CHCH_{2}Cl⟶\\ {\left(\;CH_{2}=CHCH_{2}\;\right)}_{2}N^{+}{\left(\;CH_{3}\;\right)}_{2}{Cl}^{-} \end{array}$$

In addition, there were some other side reactions. An equal amount of HCl companying with the formation of tertiary amine would be produced according to the alkylation reaction. And HCl would then react with dimethylamine immediately to form dimethylamine hydrochloride. The reaction equation was as follows:

(5) $$\begin{array}{c} {\left(\;CH_{3}\;\right)}_{2}NH+HCl⟶{\left(\;CH_{3}\;\right)}_{2}NH\cdot HCl \end{array}$$

Of course, HCl could also react with allyldimethylamine to form hydrochloride.

(6) CH2=CHCH2NCH32+HCl⟶CH2=CHCH2NCH32·HCl

Since the alkality of dimethylamine was stronger than allyldimethylamine, the formation of dimethylamine hydrochloride occupied a larger portion. Because salification of amines prohibited the nucleophilic reaction and quaternarization [25], alkali should be added to neutralize HCl and to release the two amines. The reaction equations of neutralization were as follows:

(7) $$\begin{array}{c} {\left(\;CH_{3}\;\right)}_{2}NH\cdot HCl+NaOH⟶\\ {\left(\;CH_{3}\;\right)}_{2}NH+NaCl+H_{2}O \end{array}$$

(8) $$\begin{array}{c} {CH}_{2}={CHCH}_{2}N{\left(\;{CH}_{3}\;\right)}_{2}\cdot HCl+NaOH⟶\\ {CH}_{2}={CHCH}_{2}N{\left(\;CH_{3}\;\right)}_{2}+NaCl+H_{2}O \end{array}$$

However, the allyl chloride existed together with amines in the reaction system, which would be hydrolyzed to generate allyl alcohol when OH^−^ was added.

(9) CH2=CHCH2Cl+NaOH⟶CH2=CHCH2OH+NaCl

It could be seen that the byproducts of the whole reaction process included the unreacted raw materials such as dimethylamine ((CH_3_)_2_NH) and allyl chloride (CH_2_=CHCH_2_Cl), the intermediate products such as dimethylamine hydrochloride ((CH_3_)_2_N^+^H_2_Cl), allyldimethylamine (CH_2_=CHCH_2_N(CH_3_)_2_) and its hydrochloride salt (CH_2_=CHCH_2_N^+^H(CH_3_)_2_Cl^−^), and the other byproducts such as allyl alcohol (CH_2_=CHCH_2_OH) from the hydrolyzation of allyl chloride in alkaline medium and allyl aldehyde (CH_2_=CHCHO) from oxidation of allyl alcohol.

In this paper, we particularly focused on volatile impurities which would cause undesirable inhibition and retardation effect in polymerization process.

### 3.2. Optimization of Chromatographic Conditions

#### 3.2.1. Selection of Chromatographic Columns

Considering the principle of “like dissolves likes” in column stationary phase selection together with boiling points and polarity (dipole moment) of impurities [24], three kinds of columns with increasing polarity including SE-54, PEG-20M, and alkali-modified PEG-20M were chosen for the GC analysis.

**Figure 1:**
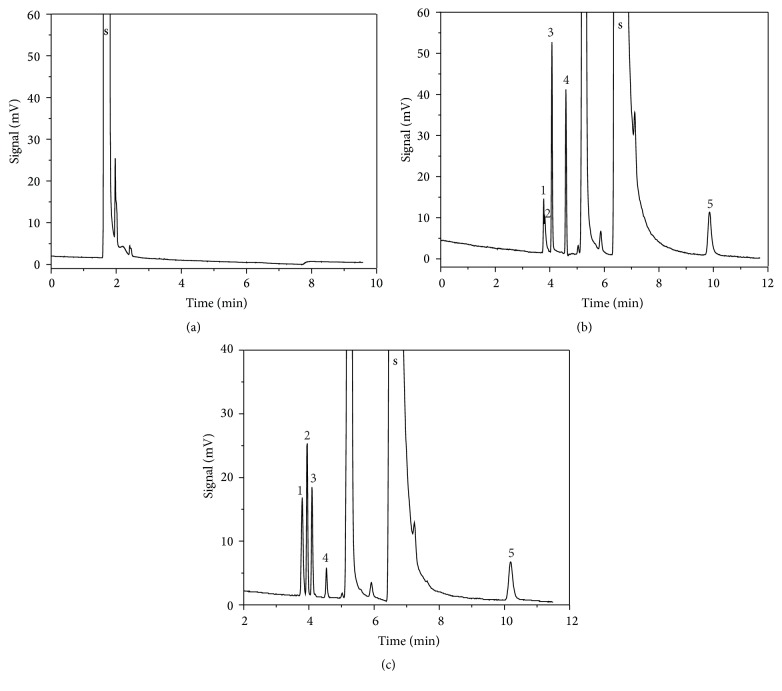
Typical chromatogram with different separation columns of SE-54 (a), PEG-20M (b) and alkali-modified PEG-20M (c). Peaks: solvent (s), dimethylamine (1), allyldimethylamine (2), allyl chloride (3), allyl aldehyde (4), and allyl alcohol (5).

For SE-54 column, impurities peaks and solvents peaks overlapped together to form only one peak even when using various solvents accompanied with adjusting GC conditions (Figure 1(a)). For PEG-20M column, the chromatogram using trichloromethane as the solvent was shown as in Figure 1(b) and the peaks of allyl chloride, allyl aldehyde, and allyl alcohol could be completely separated. However, the separation between dimethylamine and allyldimethylamine was not ideal, so the polarity of column should be adjusted to obtain a desirable separation effect of these two amines on the basis of good separation of other analytes. Therefore, an alkali-modified PEG-20M column was used in the separation. For alkali-modified PEG-20M column, the separation effect of possible volatile impurities including the two amines was enhanced clearly (Figure 1(c)). The total retention time here was very short, so it was suitable for the analysis of all the possible volatile impurities. Therefore, the modified PEG-20M column was chosen as the separation column in this paper. The polarity of dimethylamine and allyldimethylamine was close, and the retention time was almost the same, so they could not be remarkably separated by PEG-20M column. Because both of the two amines were alkaline and the alkality of dimethylamine was stronger than that of allyldimethylamine, a method of alkali modification on the PEG-20M column was applied to shorten retention time of more alkaline dimethylamine and to prolong retention time of less alkali allyldimethylamine, and the separation effect of the two amines was obviously improved.

#### 3.2.2. Determination of Analysis Conditions

**Figure 2:**
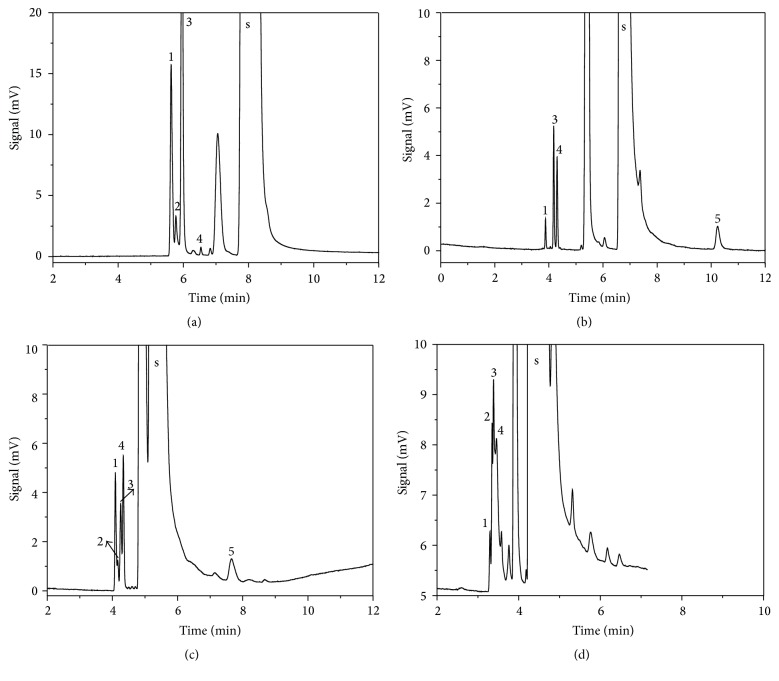
Effect of initial temperature and carrier gas flow rate of (a) 40°C and 10 mL·min^−1^, (b) 40°C and 20 mL·min^−1^, (c) 50°C and 20 mL·min^−1^, and (d) 50°C and 30 mL·min^−1^. Peaks: solvent (s), dimethylamine (1), allyldimethylamine (2), allyl chloride (3), allyl aldehyde (4), and allyl alcohol (5).

Figure 2 shows separation effect with different initial column temperature and carrier gas flow rate. Considering the boiling point range of the 5 possible volatile impurities being 6.9~96.9°C, the optimization conditions were tested by setting initial column temperatures of 40, 50, and 60°C and carrier gas flow rates of 10, 20, and 30 mL·min^−1^, respectively. Among them, the separation degree and peak shape were fine with column temperature of 40°C and flow rate of 20 mL·min^−1^ (Figure 2(b)), and the impurities could separate with each other. Usually, low temperature and low flow rate resulted in long retention time, while high temperature and high flow rate made it difficult to separate components. In 5 possible volatile impurities, the boiling point of dimethylamine was low (6.9°C) and that of allyl alcohol was high (96.9°C), and the others were 40–60°C; meanwhile there existed the effect of polarity, so the selected column temperature 40°C could satisfy the requirement of separation.

**Figure 3:**
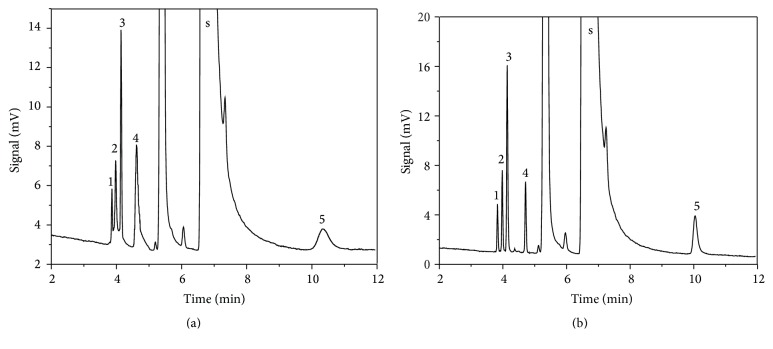
Typical chromatogram with different heating rates of (a) constant temperature and (b) 10°C·min^−1^. Peaks: solvent (s), dimethylamine (1), allyldimethylamine (2), allyl chloride (3), allyl aldehyde (4), and allyl alcohol (5).

Because of the wide boiling point range of analytes, a constant column temperature was unfavorable to multicomponent separation. Usually, a programmed temperature could make the column temperature corresponding to the boiling points of analytes, so the components with low and high boiling points could be isolated enough with suitable retention time and the peak shape was fine. Heating rates of 5, 10, 15, 20, 25°C·min^−1^ were investigated in this paper.  Figure 3 showed that the better separation effect could be obtained with heat rate of 10°C·min^−1^ by comparing the chromatograms of constant temperature; in particular the peak shape of allyl alcohol was obviously improved with programmed temperature.

### 3.3. Selection of Quantitative Methods

#### 3.3.1. Comparison of Internal and External Standard Methods

External standard method and internal standard method are commonly used quantitative methods for chromatography [26]. For the former method, the sampling condition should be paid more attention to ensuring the sample quantity precisely and parallelly. For the latter method, the key point was the selection of internal standards being stable and not overlapping with peaks of the analytes. Therefore, standard solutions of allyl chloride with high, medium, and low concentrations were detected by external and internal standard methods by GC, and the results of 5 consecutive samples were shown in Table 1. The results showed that the relative standard deviation (RSD) of peak areas (*A*_*i*_) were relatively large such as 11.58%–17.42% for allyl chloride when using external standard method, while the RSD of peak area ratios (*A*_*i*_/*A*_Standard_) were only 1.41%–2.27% when using internal standard method. The reason was that stable operating conditions, precise sampling amounts, and good repeatability were required for external standard method, and the sampling error would be accumulated and calculated into the RSD, but sampling error would not affect the RSD for internal standard method. So, the internal standard method was chosen for the analysis of impurities to ensure the accuracy of detection results.

#### 3.3.2. Selection of Internal Standards

**Figure 4:**
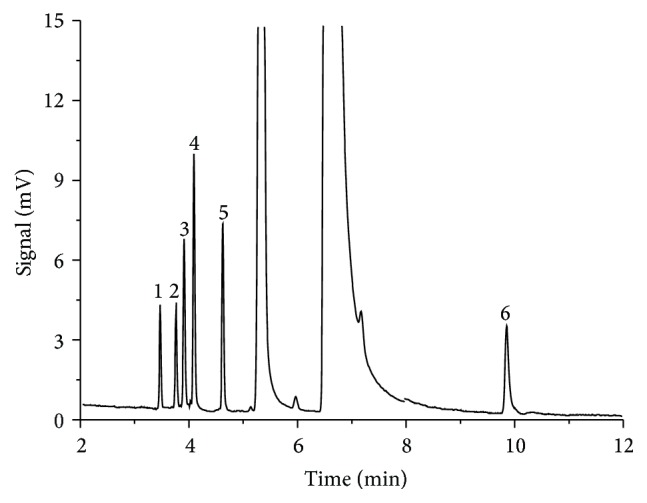
Chromatogram of internal standard and impurities (modified PEG-20M capillary column. Peaks: 1, *n*-hexane; 2, dimethylamine; 3, allyldimethylamine; 4, allyl chloride; 5, allyl aldehyde; 6, allyl alcohol).

According to the requirement of internal standard [24], several commonly used organic compounds and solvents including methanol, ethanol, propanol, isopropanol, *n*-hexane, and acetone (Table 2) were selected as the internal standards considering the boiling points and polarity of impurities and solvents. The results showed that the retention time of n-hexane was 3.46 min and it could be completely separated with impurities of allyl chloride (4.16 min), allyl aldehyde (4.68 min), allyl alcohol (9.93 min), dimethylamine (3.82 min), and allyldimethylamine (3.99 min) and extraction solvent of trichloromethane, and there were no chemical reactions between n-hexane and the analytes, so n-hexane was chosen as the internal standard. The gas chromatogram with n-hexane as internal standard and trichloromethane as extraction solvent was shown in Figure 4.

### 3.4. Optimization of Extraction

#### 3.4.1. Effect of Monomer Concentration

**Figure 5:**
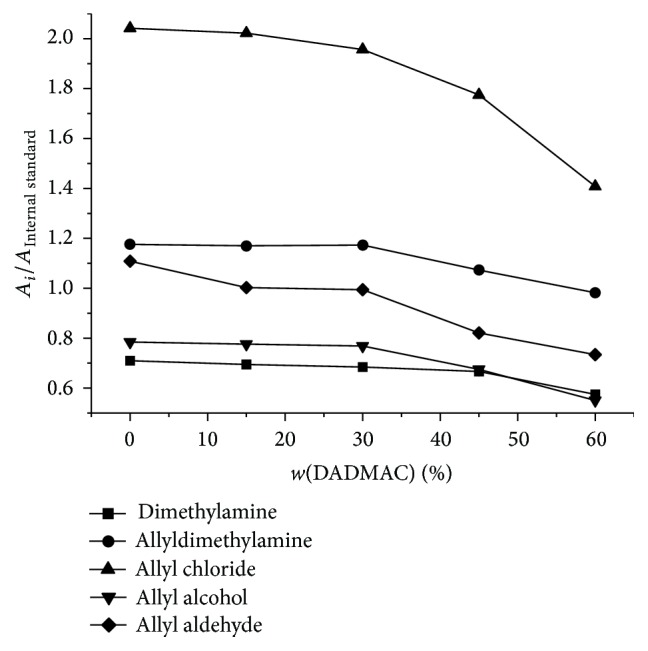
Effect of DADMAC concentration on the peaks of analytes.

The solvent extraction procedure was used to separate volatile impurities in monomer aqueous solution. However, extraction efficiency of impurities would be affected by whether the organic phase could fully be mixed with aqueous phase in extraction and then well demixed with aqueous phase after extraction [27]. As the apparent viscosity change of DADMAC monomer solution caused by changing mass fraction would have an influence on the extraction efficiency, a suitable solution concentration should be determined by experiments. Accordingly, the concentrations of DADMAC monomer solutions were adjusted to 0 wt%–60 wt% by double distilled water, while the concentration of impurities was set at 50 mg·L^−1^. Then the DADMAC samples were analyzed and the results in Figure 5 showed that, with the increase of monomer concentration, the extraction efficiency decreased gradually. The reason might be that the raising viscosity of DADMAC solution might lead to inadequate mix of extraction solvent with monomer solution and poor separation effect of them after extraction. Moreover, the change of monomer concentration would change the polarity of the aqueous layer and the distribution of impurities between organic layer and aqueous layer would be affected accordingly. However, the concentration of impurities decreased together with the decrease of DADMAC concentration, and it was also unfavorable for the quantitative accuracy. Therefore, DADMAC concentration of 30 wt% was chosen for the subsequent experiments.

#### 3.4.2. Investigation of Extraction Solvents

**Figure 6:**
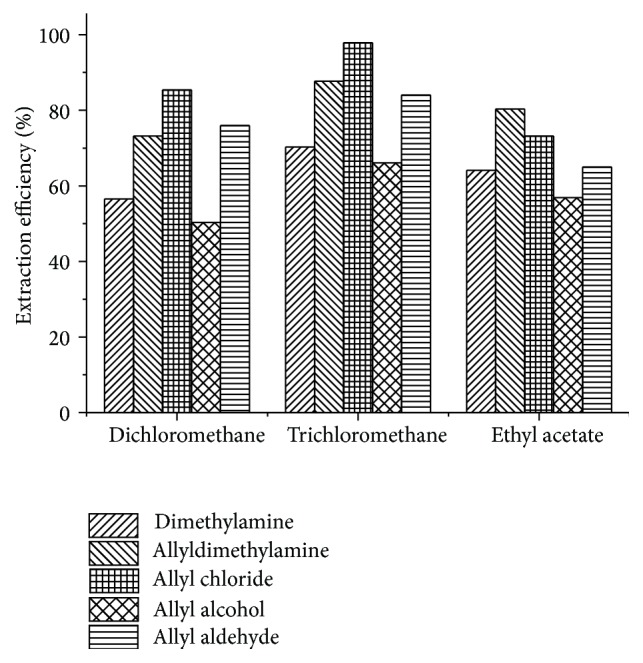
Effect of solvents on extraction efficiency.

The extraction solvent was extremely important for extraction efficiency of impurities in a quantitative method [26, 27], so the extraction solvents such as dichloroethane, trichloromethane, and ethyl acetate were further investigated for the qualitative analysis for dimethylamine and allyldimethylamine. The results as in Figure 6 showed that the extraction efficiency of the solvent trichloromethane was the highest for dimethylamine, allyldimethylamine, allyl chloride, allyl alcohol, and allyl aldehyde with the values of 70.3%, 87.7%, 97.9%, 66.1%, and 84.0%, respectively. Generally, considering the principle of “like dissolves likes,” the solubility of volatile impurities would be larger in solvents with higher polarity, namely, higher extraction efficiency. However, although the polarity of ethyl acetate was similar to that of trichloromethane (with *n*-hexane polarity of 0 as the reference, the polarity of trichloromethane was 4.4 and polarity of ethyl acetate was 4.3) [25], the water solubility of ethyl acetate (8.3 g in 100 mL water at 20°C) [25] was much higher than the latter. Therefore, a certain amount of impurities would be still left and distributed in the aqueous phase after the extraction by ethyl acetate, and the extraction efficiency was lower accordingly. Figure 6 also showed that the extraction efficiency of allyl alcohol was lower than that of the other two impurities by using all kinds of solvents, and this might be due to the high solubility of allyl alcohol in water because of the high polarity.

### 3.5. Method Validation

#### 3.5.1. Calibration and Limits of Detection (LOD)

The calibration curves were created with the serial mixed working standard solutions of impurities and constructed by plotting the peak area ratio of impurities (*A*_*i*_/*A*_Standard_) as ordinate versus the concentration of impurities as abscissa. The limit of detection (LOD) was determined as 3*S*_0_ where *S*_0_ is the standard deviation as the concentration approaches zero. The results of calibration curves and LODs in Table 2 showed good linear relationship (*R*^2^ > 0.992) in the range of 1–100 mg·L^−1^ with LODs of 0.1–0.2 mg·L^−1^.

#### 3.5.2. Precision and Recovery

Precision and recovery of the established method were evaluated with spiked samples at three concentration levels for the impurities-containing DADMAC monomer solutions. The concentration of impurities could be calculated by the peak area ratio (*A*_*i*_/*A*_Standard_) and calibration curves, and the results were listed in Table 3. From the table, it could be seen that the recoveries of dimethylamine, allyldimethylamine, allyl chloride, allyl alcohol, and allyl aldehyde were 95.6%–107.5%, 99.4%–104.1%, 95.0%–103.3%, 95.6%–98.9%, and 96.9%–99.5% with RSDs of 1.67%–5.95%, 1.59%–2.49%, 1.45%–2.21%, 1.97%–3.75%, and 1.40%–2.05%, respectively. The established method showed good precisions and recoveries for all three impurity compounds despite the three different concentration levels, and it could entirely meet the needs of precisely quantitative analysis for them.

### 3.6. Analysis of DADMAC Samples

Some self-made DADMAC samples in lab and in pilot plants and the commercial DADMAC samples from representative companies in China were chosen to analyze the contents of volatile impurities. The results in Table 4 showed that the contents of these impurities in DADMAC samples were different from each other. The molecular weights (calculated by intrinsic viscosity [*η*]) of poly-DADMACs changed with the variation of the sorts of impurities and their contents even under the same optimum polymerization condition [14], so that it could further not only prove the obvious effect of the impurities on the molecular weight of polymer products, but also illustrate the importance to establish a product quality standard for promoting the overall level of the DADMAC production in industry.

## 4. Conclusions

A novel method of GC coupled with solvent extraction for the quantitative analysis of volatile impurities in DADMAC monomer solution was established with trichloromethane as extraction solvent and n-hexane as internal standard. The results showed excellent linear relationships of 5 volatile impurities (dimethylamine, allyldimethylamine, allyl chloride, allyl alcohol, and allyl aldehyde) in the range of 1–100 mg·L^−1^, together with good specificity, recovery (95.0%–107.5%), and relative standard deviation (RSD, 1.40%–7.67%). Finally, several DADMAC samples were analyzed successfully using this method and a satisfactory result was obtained. The established quantitative determination method not only can have industrial application value because of simplicity, speediness, and accuracy, but also can provide a foundation for the establishment of DADMAC product quality standard and the research on the regulation and mechanism concerning the effect of impurities on DADMAC polymerization reactivity.

## Figures and Tables

**Table 1:** Comparison of external and internal standard methods.

Methods	Conc. (mg L^−1^)	Found	RSD (%)
External standard method (peak area, *A*_*i*_)	10	938 854 1305 1042 1202	17.35
	30	2741 2983 3364 3687 3051	11.58
	50	3801 6132 5349 5895 5859	17.42

Internal standard method (peak area ratio, *A*_*i*_/*A*_Standard_)	10	0.419 0.413 0.412 0.427 0.420	1.45
	30	1.25 1.24 1.30 1.29 1.24	2.27
	50	2.21 2.14 2.15 2.19 2.16	1.41

**Table 2:** Calibration curve and LODs for allyl chloride and its derivatives.

Impurities	Concentration range (mg L^−1^)	Equation	*R* ^2^	LOD (mg L^−1^)
Dimethylamine	1–100	*y* = 0.0283*x* − 0.1581	0.9974	0.2
Allyldimethylamine	1–100	*y* = 0.0270*x* − 0.0080	0.9966	0.1
Allyl chloride	1–100	*y* = 0.0436*x* + 0.0075	0.9976	0.1
Allyl alcohol	1–100	*y* = 0.0417*x* + 0.0586	0.9942	0.1
Allyl aldehyde	1–100	*y* = 0.0179*x* − 0.0673	0.9924	0.2

**Table 3:** Precision and recovery for allyl chloride and its derivatives (*n* = 6).

Impurities	Initial found (mg L^−1^)	Added (mg L^−1^)	Total found (mg L^−1^)	Recovery (%)	RSD (%)
Dimethylamine	10.75	10.0	22.30	107.5	5.95
	19.12	20.0	37.39	95.6	3.77
	27.42	30.0	56.21	97.9	1.67

Allyldimethylamine	24.85	25.0	49.55	99.4	1.59
	32.44	30.0	64.68	103.6	1.74
	41.59	40.0	84.93	104.1	2.49

Allyl chloride	12.39	10.0	22.23	99.3	1.62
	19.00	20.0	37.05	95.0	1.45
	26.80	30.0	58.67	103.3	2.21

Allyl alcohol	11.71	10.0	21.47	98.9	2.15
	16.24	20.0	34.64	95.6	3.75
	24.73	30.0	53.42	97.6	1.97

Allyl aldehyde	24.88	10.0	33.97	97.4	1.40
	38.77	20.0	56.95	96.9	2.05
	53.58	30.0	83.16	99.5	1.73

**Table 4:** Analytical results for several real DADMAC samples.

Samples	Impurities contents (mg L^−1^)					[*η*] (dL g^−1^)
	Dimethylamine	Allyldimethylamine	Allyl chloride	Allyl aldehyde	Allyl alcohol	
50 g grade lab experiment	184.96	407.22	45.82	1305.18	85.92	unreactive
50 kg grade pilot test P6#	ND	ND	ND	ND	ND	3.17
500 kg grade pilot test AZ#	ND	47.51	63.75	ND	ND	0.43
Jiangsu Feymer Technology Co., Ltd.	ND	ND	ND	ND	ND	2.31
Shandong Luyue Chemical Co., Ltd.	ND	147.33	17.04	4.96	ND	0.88
Shandong Polymer Bio-Chemicals Co., Ltd.	ND	29.24	ND	ND	ND	1.84
Hangzhou Yinhu Chemical Co., Ltd.	272.06	375.45	118.15	74.29	ND	0.13

ND = not detected.
